# Effect of Wetting Characteristics of Polishing Fluid on the Quality of Water-Dissolution Polishing of KDP Crystals

**DOI:** 10.3390/mi13040535

**Published:** 2022-03-29

**Authors:** Xu Wang, Hang Gao, Qianfa Deng, Jinhu Wang, Hongyu Chen, Julong Yuan

**Affiliations:** 1 Ultra-Precision Machining Center, Key Laboratory of Special Purpose Equipment and Advanced Processing Technology of Ministry of Education, College of Mechanical Engineering, Zhejiang University of Technology, Hangzhou 310024, China; qfdeng@zjut.edu.cn (Q.D.); wangjinhu@zjut.edu.cn (J.W.); hychen@zjut.edu.cn (H.C.); jlyuan@zjut.edu.cn (J.Y.); 2 Key Laboratory for Precision and Non-Traditional Machining Technology of Ministry of Education, Dalian University of Technology, Dalian 116024, China

**Keywords:** KDP crystals, water-dissolution polishing, wetting characteristics, surface quality

## Abstract

KDP crystals constitute the only laser-frequency conversion and electro-optical switches that can be used in laser systems for inertial confinement fusion. However, KDP crystals are difficult to produce because of their inherent softness, brittleness, water-solubility, and temperature sensitivity. The authors’ group developed a water-dissolution polishing method in previous studies to obtain near-damage-free KDP surfaces. In this article, the effect of the wetting characteristics of the water dissolution polishing fluid on the crystal surface—a factor rarely considered in the usual process optimization—on the polished surface quality was comprehensively studied. The mean radius of micro water droplets at 5 wt.% and 7.5 wt.% water content was approximately 0.6 nm and 1.2 nm, respectively. Theoretically, the smaller micro water droplet size is beneficial to the polished surface quality. When the water content was 5 wt.%, due to the poor wetting characteristics of the polishing fluid, surface scratches appeared on the polished surface; when the water content was 7.5 wt.%, the effects of the wetting characteristics and the radius of the micro water droplets reached a balance, and the polished surface quality was the best (Ra 1.260 nm). These results confirm that the wetting characteristics of the polishing fluid constitute one of the key factors that must be considered. This study proves that the wetting characteristics of the polishing fluid should be improved during the optimization process of polishing fluid composition when using oil-based polishing fluids for ultra-precision polishing.

## 1. Introduction

Potassium dihydrogen phosphate (KH_2_PO_4_ and KDP) crystals have excellent nonlinear electro-optical properties and currently constitute the only material that can be used as electro-optical switching and frequency doubling conversion elements in laser-induced inertial confinement fusion (ICF) technology [1]. High-power ICF laser devices require a high number of large-size, ultra-high-quality KDP crystal elements. For instance, this is the case of the National Ignition Facility [2,3], which requires a large diameter-thickness ratio (410 mm × 410 mm × 10 mm), high surface quality (surface roughness root mean square (rms) ≤ 5 nm, transmission wave-front aberration less or equal than λ/6 PV, and high laser-induced damage threshold (≥15 J/cm ^2^) for more than 600 pieces of KDP components. KDP crystals are materials that are extremely difficult to process owing to their soft and brittle nature, strong anisotropy, and extreme sensitivity to processing temperature [4]. Moreover, KDP crystals are soluble in water (solubility of 33 g/100 g H_2_O at 25 °C) [5], and prolonged exposure to air will absorb moisture and damage the processed surface, further increasing the difficulty of ultra-precision processing and storage of KDP crystals.

Researchers have explored multiple techniques to process KDP crystals. Single-point diamond turning is the most important technique to process KDP components in engineering, producing KDP crystals with a high surface quality. However, micro-waviness appears on the surface of these crystals [6,7]. Such micro-waviness can have an impact on the laser-induced damage threshold of the KDP samples [8]. Moreover, the mechanical processing can produce subsurface damage that affects the optical performance of the KDP elements [9,10].

Ultra-precision grinding is also a commonly used technique in the processing of large-size optical components [11,12]. Qu et al. [13,14] successfully applied an ultra-precision grinding method to obtain ultra-smooth KDP crystal surfaces. Yin et al. [15] developed a magnetorheological polishing technique with controlled fluid-crystal temperature difference to improve the polishing quality and efficiency. Shi et al. [16] developed a combined magnetorheological and ion beam polishing treatment to be applied on the surface after fly-cutting. The ion beam was used to remove residual iron ions from the surface after magnetorheological polishing, which increased the crystal laser-induced damage threshold. Gao et al. [17] proposed an abrasive-free jet polishing method for KDP crystals to address surface particle residues and thermal effects. Despite the significant progress achieved, problems such as abrasive grain embedding, surface atomization, iron powder embedding, and high cost remain. Ultra-precision processing of large-size KDP crystal elements is still one of the limitations of ICF engineering.

The research group authoring this paper developed an oil-based, abrasive-free polishing fluid with an “water-in-oil” structure exploiting the water-soluble nature of KDP crystals, and verified the feasibility of this water-soluble processing method by obtaining a non-destructive and ultra-smooth surface via a continuous polishing method only through the physical dissolution of micro water droplets in the polishing fluid [18]. A flattened polishing with surface roughness rms of 2.182 nm and flatness of 22.013 μm (KDP crystal size of 100 mm × 100 mm × 10 mm) was achieved using an indefinite eccentric motion [19]. Meanwhile, in combination with computer-controlled optical surfacing technology, which is widely employed for large-size optical component processing, a tool influencing the function of the water-dissolution numerical control polishing method, was implemented by using small tools to process large-size KDP crystals [20]. Thus, the micro waviness on the surface after single-point diamond turning processing was successfully removed, reducing the surface roughness rms to values below 3 nm [21]. The laser-induced damage thresholds of KDP crystals after processing by different methods were also compared, and the morphology of laser damage points was analyzed. It was concluded that the proposed water-dissolution polishing could enhance the laser-induced damage thresholds of KDP crystals [22].

In a previous study, the effect of water content of oil-based water-dissolution polishing fluid on the surface roughness of KDP crystals was also studied. We found that the surface roughness after polishing does not always decrease with the decrease of the water content of polishing fluid, and that the surface roughness becomes worse when the water content of polishing fluid is too low (5 wt.%). However, theoretically, the lower the water content is, the smaller the size of micro water droplets in the polishing fluid becomes, and the better the surface quality should be. In addition, we also experimentally found that there is a significant difference in the wetting characteristics of the crystal surface among polishing fluids with different water contents. Furthermore, the effect of wetting characteristics on the quality of ultra-precision polishing has been rarely reported.

To analyze the factors affecting the quality of KDP water-dissolution polishing, the effect of the wetting characteristics of polishing fluid on the crystal surface—a factor rarely considered in the usual process optimization—on the polishing quality was investigated in the current study.

## 2. Correlation between Surface Roughness and Water Content during the Water-Dissolution Polishing Process of KDP Crystals

### 2.1. Selective Removal Mechanism of Water-Dissolution Polishing

**Figure 1:**
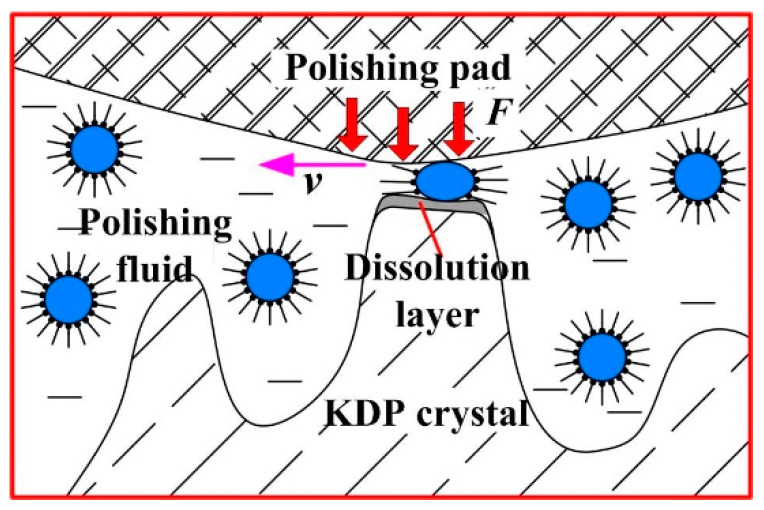
Illustration of the selective removal of material from a KDP crystal.

The selective removal mechanism of water-dissolution, ultra-precision polishing of KDP crystals that can produce ultra-smooth surfaces is shown in Figure 1. In the oil-based polishing fluid used, the micro water droplets are wrapped in surfactants and dispersed in the oil-phase mother liquor to form a “water-in-oil” structure. In the polishing area, the polishing pad is in contact with the crystal surface material under a certain pressure. Under such a mechanical action, the “water-in-oil” structure at the rough peak of the polishing pad and the crystal surface are squeezed and deformed by friction, and the crystal material at the high point is dissolved and removed by the released water. The dissolved products are then carried away from the crystal surface by the mechanical action of the polishing pad and the flowing action of the polishing fluid, while at the low concave part of the crystal surface, the polishing pad is not in direct contact with the crystal material, and the “water-in-oil” structure is in a stable state, so no dissolution phenomenon occurs. This achieves selective removal of the crystal surface roughness peak and reduces the crystal surface roughness [18].

### 2.2. Analysis of the Effect of Water Content of Water-Dissolution Polishing Fluid on the Polished Surface Roughness

**Figure 2:**
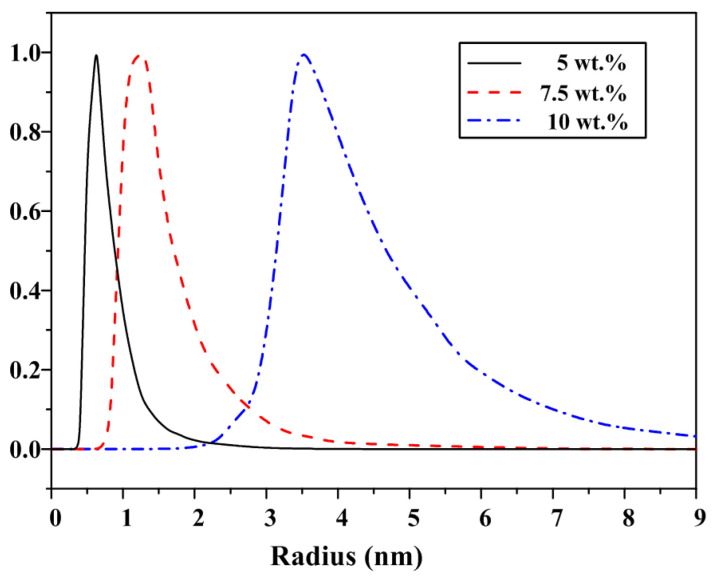
Radius distribution curve of micro water droplets in different polishing fluids.

The size of the micro water droplets in the “water-in-oil” polishing fluid is directly related to the polished surface roughness. If the size of the micro water droplets is too large, the ultra-smooth surface with nanoscale roughness will not be formed. The micro water droplet size in the polishing fluid with different water contents was measured by a dynamic light scattering (DLS) instrument (model no.: ALV-NIBS); the results are shown in Figure 2. The micro water droplet radius was basically distributed within 10 nm, which proves the feasibility of the above described removal mechanism. Note also that the micro water droplet size in polishing fluid increases with the increase of water content in the polishing fluid. The average values of micro water droplet radius when the polishing fluid contained 5 wt.%, 7.5 wt.%, and 10 wt.% water were approximately 0.6 nm, 1.2 nm, and 3.8 nm, respectively. Theoretically, the lower the water content of the polishing fluid, the smaller the size of the micro water droplets—which should be more favorable for the selective removal of crystal surface roughness peaks—and the lower the surface roughness after polishing.

**Figure 3:**
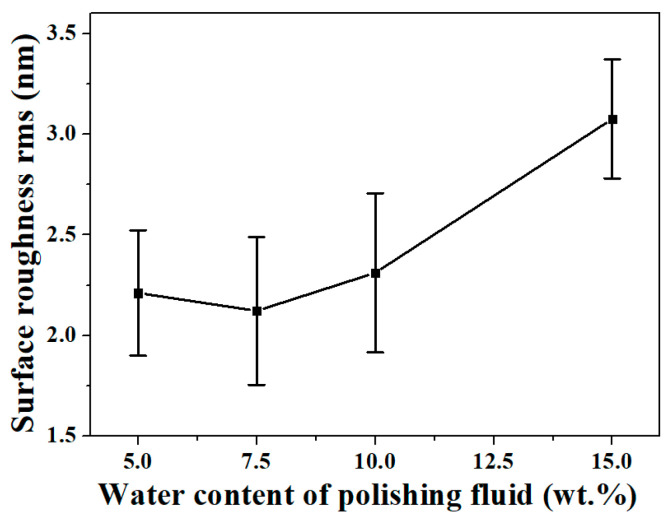
Variations in surface roughness with water content of polishing fluid (the data used to plot this curve are from [21]).

In a previous study, we analyzed the variation pattern of the surface roughness of KDP crystals after polishing with the water content of the water-dissolution polishing fluid (as shown in Figure 3). When the water content was greater than 7.5 wt.%, the surface roughness decreased significantly with the decrease of water content, as expected. However, when the water content was reduced to 5 wt.%, the surface roughness value after polishing was higher than that at 7.5 wt.% [21], and it did not keep decreasing with the decrease in water content, which was inconsistent with the theoretical analysis based on the micro water droplet size.

## 3. Analysis of the Effect of Polishing Fluid Viscosity on the Surface Quality of Water-Dissolution Polishing

In the traditional polishing process, it is generally assumed that a lower viscosity of the polishing fluid will be beneficial to obtain a higher quality surface. Gutmann et al. [23] considered that a high viscosity polishing fluid cannot be easily transported to the processing area between the polishing pad and the polished surface compared to a low viscosity polishing fluid, which will lead to a lubrication state in the polished area that is not conducive to processing and affects the quality of the polished surface, with defects such as scratches possibly occurring. Mullany et al. [24] assumed that a lower viscosity of polishing fluid leads to a higher friction, which can improve the material removal rate of polishing, and is conducive to obtaining an ultra-smooth surface.

**Figure 4:**
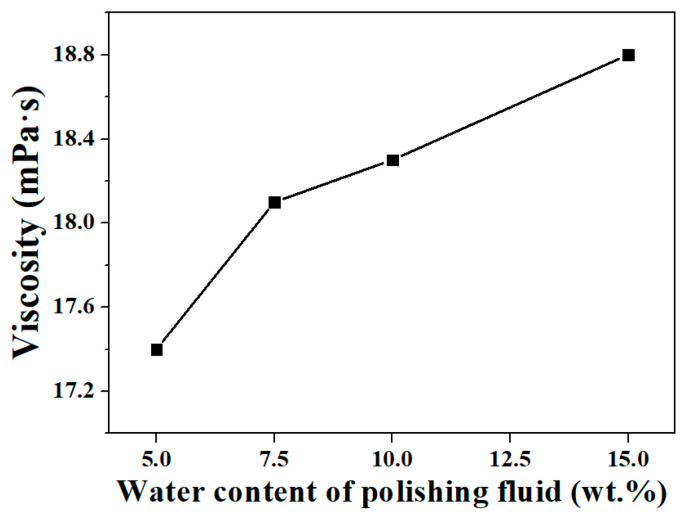
Variations in the viscosity of polishing fluid with water content.

Changes in the water content of the polishing fluid during the water-dissolution polishing of KDP crystals can also cause changes in viscosity. In this study, an NDJ-1 rotary viscometer was used to measure the viscosity of a polishing fluid with different levels of water content. The measurement results are shown in Figure 4. The viscosity of the polishing fluid increased with the increase in water content. According to the results of Gutmann, Mullany, and others, the viscosity of polishing fluid is lower when the water content is lower, which should theoretically facilitate the processing, but is not consistent with the actual results when KDP crystals are processed by water-dissolution ultra-precision polishing methods. Therefore, the difference in viscosity is not the reason for the degradation in polished surface quality when the water content is too low.

## 4. Analysis of the Effect of the Wetting Characteristics of Polishing Fluid on the Surface Quality of Water-Dissolution Polishing

**Figure 5:**
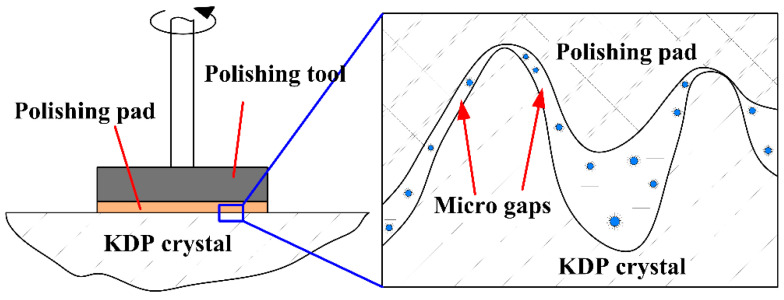
Illustration of micro gaps between polishing pad and KDP crystal.

In actual processing, the surface of KDP crystals and a polishing pad with a certain degree of elasticity do not perfectly fit each other. A large number of small-size gaps exists between the rough peak of the crystal surface and the polishing pad (as shown in Figure 5). Selective localized water-dissolution at the rough peaks of the crystal surface can only occur when the polishing fluid is able to enter these gaps sufficiently. During the polishing process, if the polishing fluid cannot be guaranteed to fully enter the small-size gaps between the polishing pad and the KDP crystal, the rough peak will lack sufficient polishing fluid to ensure that material removal occurs. Thus, even direct contact friction between the polishing pad and the crystal surface will occur locally, which will have a negative effect on the quality of the polished surface and directly affect the acquisition of an ultra-smooth surface.

Given that the size of the rough peak on the KDP crystal surface is extremely small (micro-nanometer scale), the gaps between the polishing pad and the surface can be approximated as micro-nanometer scale pipes, demanding the consideration of the effect of fluid capillarity. To evaluate the ability of the polishing fluid to enter the pore space between the polishing pad and the crystal surface, denoted as *E_a_*, with reference to the formula for calculation of the column rise height *h* of the fluid in the capillary, *E_a_* can be considered to be positively correlated with *h*:

(1) $$E_{a}\propto h=\frac{2\gamma \cos\theta }{\rho gr}$$

where γ is the surface tension, θ is the contact angle, *ρ* is the density of the fluid, and *r* is the radius of the capillary. Therefore, to analyze *E_a_* with a certain pore size, the surface tension, contact angle, and density of the polishing fluid need to be examined and analyzed.

**Figure 6:**
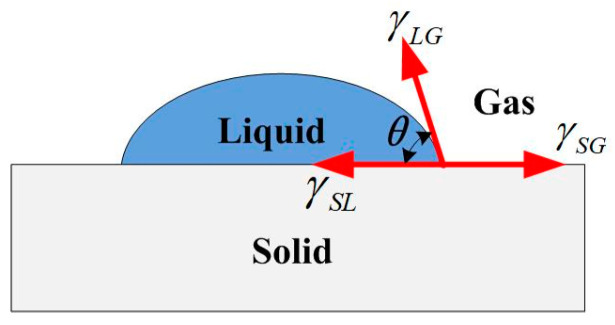
Illustration of the contact angle.

The surface of the polyurethane polishing pad was covered with pores and channels. It provided a certain storage capacity for polishing fluid, which could quickly penetrate into the surface layer of the polishing pad. Thus, the contact angle of the polishing fluid on the surface of the polishing pad can be approximated to be close to 0°. Therefore, in the present study, we assumed that the contact angle of the water-dissolution polishing fluid on the surface of KDP crystal was the main parameter impacting *E_a_*. The contact angle can be a direct measurement of the wettability of a fluid on a solid surface, i.e., the ability and tendency of a fluid to spread out on a solid surface. It is the angle between the tangent line of the liquid–gas interface and the solid–liquid interface at the junction of the solid–liquid–gas phase (as shown in Figure 6). The contact angle satisfies the Young equation:

(2) $$\cos\theta =(\gamma_{SG}-\gamma_{SL})/\gamma_{LG}$$

where $\gamma_{LG}$ is the gas–liquid interfacial tension, $\gamma_{SG}$ is the solid–gas interfacial tension, and $\gamma_{SL}$ is the solid–liquid interfacial tension. When *θ* = 0°, the liquid can completely wet the solid surface (the contact angle of ethanol on the surface of KDP crystal is 0°); when *θ* < 90°, the liquid can wet the solid surface; and when *θ* > 90°, the solid surface is hydrophobic, and the liquid cannot wet the solid surface.

If the contact angle of the polishing fluid on the surface of a KDP crystal is large, the polishing fluid will not easily enter the processing area between the polishing pad and the crystal. Therefore, to evaluate the wettability of the polishing fluid with different water contents on the surface of KDP crystals, the contact angle was measured. The surface roughness of the KDP crystal in the test was 3–5 nm, which was close to the surface state in the ultra-precision polishing process; the droplet volume of the polishing fluid was 5 μL to avoid the influence of the droplet’s self-weight on the contact angle.

**Figure 7:**
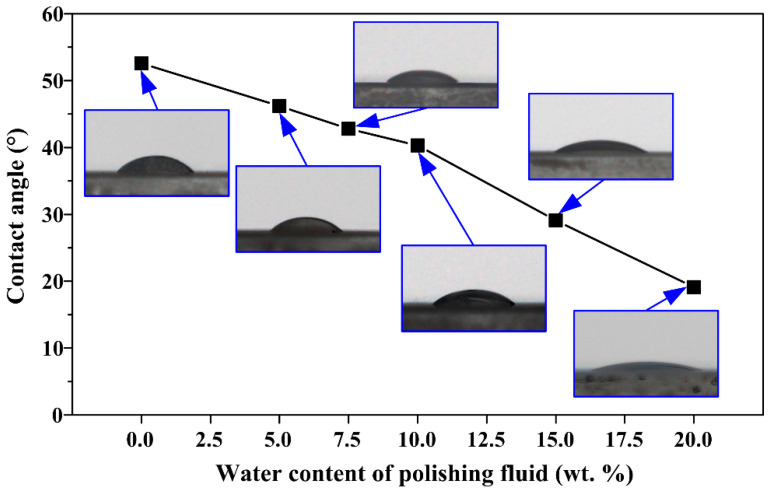
Contact angles of polishing fluid with different water contents on the KDP surface.

The test results of the contact angles of polishing fluid on the KDP surface are shown in Figure 7. The wettability of the polishing fluid on the KDP surface was the worst when the water content was 0 wt.%, and the contact angle reached 52.6°, but it could still wet the KDP crystal surface. The contact angle was 46.2° when the water content of polishing fluid was 5 wt.%. The contact angle of polishing fluid on the surface of the KDP crystal decreased with the increase of water content, and the decreasing trend was linear. When the water content increased to 20 wt.%, the contact angle decreased to 19.1°.

According to Equation (1), in addition to the contact angle, the polishing fluid density and surface tension are also important factors affecting its access to the polishing area. In this study, we used a DSA100 surface tension meter to measure the surface tension of polishing fluids with different water contents through the “hanging drop method”. Therefore, the surface tension was determined by the radius of curvature of the bottom of the hanging drop and the shape factor of the droplet when the droplet reached static-force (tension to gravity) equilibrium. The surface tension of polishing fluids with different water contents is listed in Table 1.

The surface tension of water at room temperature was 72.7 mN/m, and the surface tension of other organic components of the polishing fluid was approximately 10–40 mN/m. Note from Table 1 that only a very small increase of the surface tension of polishing fluid occurred with the increase of water content; this tension was basically maintained at approximately 25 mN/m; when the water content increased from 0% to 20%, the density of the polishing solution slightly increased from 0.82 g/cm^3^ to 0.887 g/cm^3^. The changes in contact angle were then relatively large. The variation of the value of cos*θ* was much greater than that of γ and *ρ*. Thus, it can be concluded that the surface tension and density have much less influence on the ability of the polishing fluid to enter the pore space between the polishing pad and the crystal surface than the contact angle.

Substituting the experimentally measured contact angle, surface tension, and density into Equation (1), we can easily conclude that the lower the water content of the polishing fluid, the worse its ability to enter the pore space between the polishing pad and the crystal surface. This means that in actual water-dissolution polishing, the use of polishing micro-emulsions with a too low water content will lead to the polishing fluid to enter the processing area with some difficulty, and even with local “unlubrication-friction“ of the polishing pad and crystal, the surface quality of the polished KDP crystal will be affected.

In addition to *E_a_*, the wetting work reflects the firmness of the solid–liquid interfacial bonding, and can also characterize the ability of polishing fluid to wet the KDP crystal surface. The wetting work *W_a_* is calculated according to Equation (3). It can be concluded that the larger its value, the stronger the solid–liquid interfacial bonding, and the easier to wet the solid surface. Table 2 lists the wetting work *W_a_* calculated according to the results of surface tension and contact angle measurements. It is evident that *W _a_* increases basically linearly with the increase of water content of polishing fluid, which is consistent with the variation trend of *E_a_*. This further proves that the water content of the water-dissolution polishing fluid directly affects the wettability of the processed surface, thus affecting the polishing quality.

(3) $$W_{a}=(\gamma_{SG}+\gamma_{LG})-\gamma_{SL}=\gamma_{LG}(1+\cos\theta )$$

**Figure 8:**
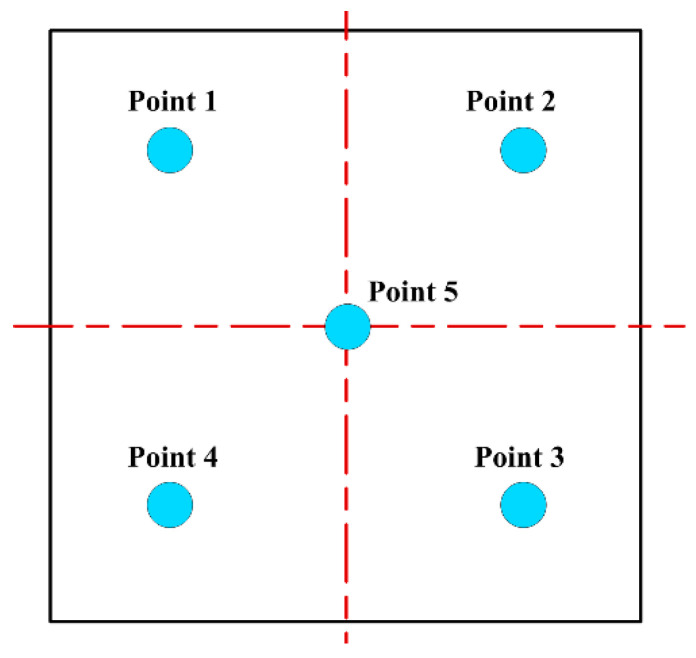
Schematic showing the five observing points on the KDP crystal.

To verify the above analysis, the surface of KDP crystals polished with a polishing fluid containing 7.5 wt.% water was polished for 20 min with a polishing fluid containing 5 wt.% water. To ensure that the observations of the polished surface were representative, five points on the KDP surface were observed with an optical microscope (Olympus MX40, Tokyo, Japan), as shown in Figure 8.

For the water-dissolution polishing method, the material is removed by water dissolution with micro water droplets in the polishing fluid, and a super smooth surface without scratches was obtained by polishing with a polishing fluid containing 7.5 wt.% water (as shown in Table 3). Under the condition of sufficient supply of polishing fluid, the surface quality of the polished KDP with polishing fluid containing 5 wt.% water crystal was not further improved. Moreover, microscopic scratches appeared locally on the crystal surface that caused damage to the polished surface.

**Figure 9:**
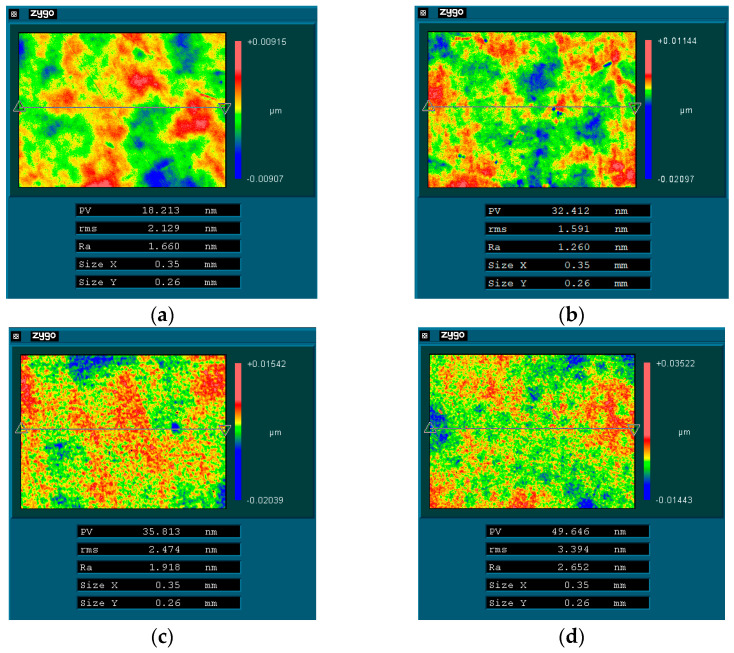
Surface roughness of KDP crystal after polishing with different water contents of polishing fluid: (**a**) 5 wt.%, (**b**) 7.5 wt.%, (**c**) 10 wt.%, and (**d**) 15 wt.%.

The surface roughness of KDP crystal was measured with a three-dimensional profilometer (ZYGO Newview 5022, Middlefield, CT, USA) over an area of 0.35 × 0.26 mm (the measuring results are shown in Figure 9). When the water content was greater than 7.5 wt.%, the surface roughness decreased significantly with the decrease in water content. The surface roughness value was Ra 1.918 nm and Ra 2.652 nm after being processed with a polishing fluid with water content of 10 wt.% and 15 wt.%, respectively. The surface roughness value decreased to Ra 1.260 nm after being processed with a polishing fluid with water content of 7.5 wt.%, while the surface roughness value increased to Ra 1.660 nm after being processed with a polishing fluid with water content of 5 wt.%. When the water content was lower than 7.5 wt.%, continuing to reduce the water content of the polishing fluid would lead to a decrease in surface quality.

These experimental results are consistent with the previous analysis. When using oil-based polishing fluid, if the wettability of the polishing fluid on the surface of the workpiece is too low, a significantly negative impact on the polishing quality is caused. The polished surface quality of KDP crystals was jointly determined by the micro water droplet size and the wetting characteristics of the polishing fluid on the KDP surface. The wettability of the polishing liquid on the KDP surface was enhanced with the increase in water content, and it was easier for the water-dissolution polishing fluid with high water content to enter the micro-porosity between the polishing pad and the crystal surface. However, when the water content was greater than 7.5 wt.%, the size of micro water droplets in the polishing fluid also increased substantially, thereby departing from achieving a high-quality surface. At this time, the surface quality after polishing still decreased with the decrease of water content. However, when the water content was reduced to 5 wt.%, the surface quality deteriorated due to the reduced wettability of the polishing solution on the KDP surface, even though the mean micro water droplet radius was reduced from 1.2 nm at 7.5 wt.% to 0.6 nm. When the water content was 7.5 wt.%, the wetting characteristics of the polishing fluid and the effect of the micro water droplet radius reached a balance, and the surface quality was optimized after polishing.

## 5. Conclusions

When using a “water-in-oil” polishing fluid for ultra-precision polishing of KDP crystals, theoretically, the lower the water content, the easier to obtain lower surface roughness. In this study, we analyzed the anomalous phenomenon that the surface quality decreases when the water content of the polishing fluid is too low in the process of water-dissolution polishing of KDP crystals. The conclusions are as follows:

1. The radius distribution of micro water droplets in water-dissolution polishing fluid was within 10 nm, and it increased significantly with the increase in water content. The mean value of the micro water droplet radius was approximately 0.6 nm when the polishing fluid contained 5 wt.% of water, it was approximately 1.2 nm with a water content of 7.5 wt.%, and it was approximately 3.8 nm when the water content was 10 wt.%.
2. The viscosity of the polishing fluid increased with the increase of water content; theoretically, a lower viscosity should be more conducive to polishing the surface quality. Thus, the polishing fluid viscosity was not considered as a key factor affecting the quality of water-dissolution polishing of KDP crystals.
3. By measuring the contact angle and surface tension, it was concluded that the wetting characteristics of the polishing solution on the crystal surface were significantly affected by the water content. The higher the water content, the better the polishing fluid wet the crystal surface, and the easier to enter the micro-porosity between the polishing pad and the crystal surface, which is theoretically beneficial to obtain a high-quality surface.
4. The surface roughness value of KDP was Ra 1.260 nm after being processed with a polishing fluid containing 7.5 wt.% water, while the surface roughness value increased to Ra 1.660 nm after being processed with a polishing fluid with a water content of 5 wt.%, and the surface became clearly scratched. Concerning the KDP crystal water-dissolution polishing method, a low water content of the polishing solution led to a decrease in the wettability, making it difficult to enter the gaps between the polishing pad and the crystal surface, and thus leading to a degradation in the quality of the polished surface. The polished surface quality of KDP crystals was jointly determined by the micro water droplet size and the wetting characteristics of the polishing fluid on the KDP surface. When the water content was 7.5 wt.%, the wetting characteristics of the polishing fluid and the effect of micro water droplet radius were balanced and the best polished surface quality was achieved.
5. When using oil-based polishing fluids for ultra-precision processing, in addition to conventional factors such as viscosity, size of polishing particles (abrasive particles, microdroplets, etc.), and polishing speed, the wetting characteristics of the polishing fluid on the processed surface constituted one of the key factors that must be considered. In the future, we will further optimize the polishing fluid composition and incorporate appropriate additives to obtain a water-dissolution polishing fluid with good microemulsion radius and wetting characteristics to further improve the surface quality of KDP crystals after polishing. The present study also proved that the wetting characteristics of the polishing fluid should be improved during the optimization process of polishing fluid composition when using oil-based polishing fluids for ultra-precision polishing.

## Figures and Tables

**Table 1:** Surface tension of polishing fluids with different water contents.

Water Content	Measuring Results	Density (g/cm^3^)	Surface Tension (mN/m)
0 wt.%	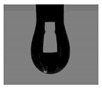	0.820	24.78
5 wt.%	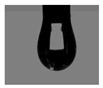	0.842	24.90
7.5 wt.%	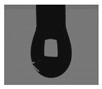	0.849	25.02
10 wt.%	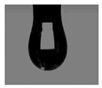	0.856	25.29
15 wt.%	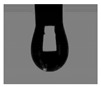	0.867	25.58
20 wt.%	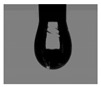	0.887	25.73

**Table 2:** Wetting work of polishing fluids with different water contents.

Water Content	Contact Angle (°)	Surface Tension (mN/m)	Wetting Work (mN/m)
0 wt.%	52.6	24.78	39.83
5 wt.%	46.2	24.90	42.13
7.5 wt.%	42.8	25.02	43.38
10 wt.%	40.3	25.29	44.58
15 wt.%	29.1	25.58	47.93
20 wt.%	19.1	25.73	50.04

**Table 3:** KDP surface after polishing with different water contents of polishing fluid.

	5 wt.%	7.5 wt.%
Point 1	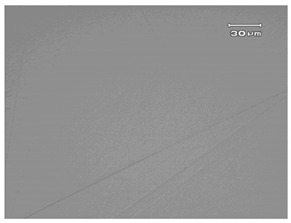	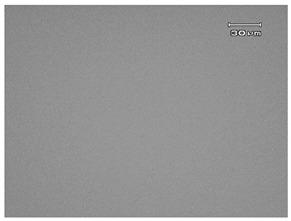
Point 2	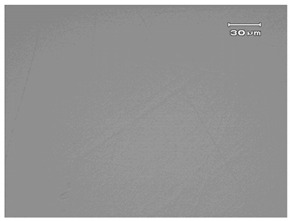	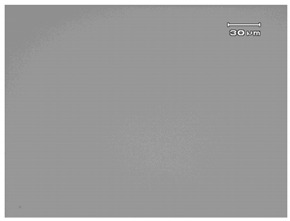
Point 3	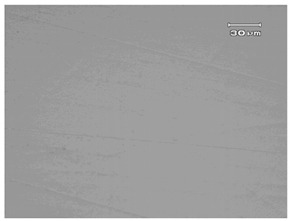	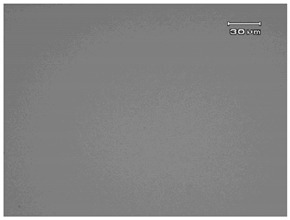
Point 4	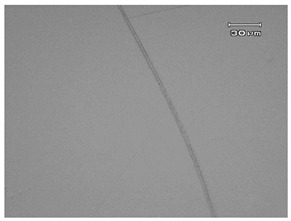	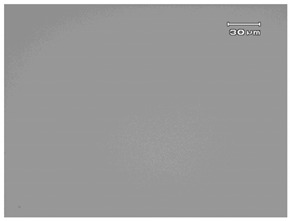
Point 5	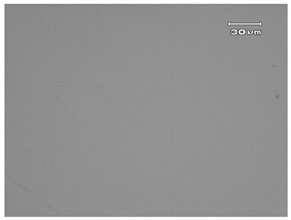	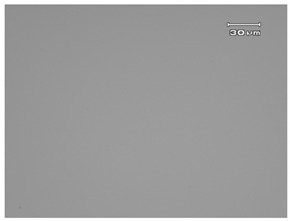

## References

[1] Zhang Z., Wang H., Quan X., Pei G., Tian M., Liu T., Long K., Li P., Rong Y. (2019). Optomechanical analysis and performance optimization of large-aperture KDP frequency converter. Opt. Laser Technol..

[2] Zylstra A.B., Nora R., Patel P., Hurricane O. (2021). Model validation for inferred Hot-Spot conditions in National Ignition Facility experiments. Phys. Plasmas.

[3] Lindl J.D., Atherton L., Amednt P., Batha S. (2011). Progress towards ignition on the National Ignition Facility. Nucl. Fusion.

[4] Hang W., Wei L., Debela T., Chen H., Zhou L., Yuan J., Ma Y. (2022). Crystallographic orientation effect on the polishing behavior of LiTaO_3_ single crystal and its correlation with strain rate sensitivity. Ceram. Int..

[5] Zhou X., Zheng W., Xu D., Luo T., Zhang Z., Wang X. (2020). Solubility measurement and thermodynamics modelling for potassium dihydrogen phosphate in a water-ethanol system from 293.2 to 323.2 K. Fluid Phase Equil..

[6] Chen D., Zhang S., Liu J., Zha C., Pan R. (2020). Morphological analysis of KDP-crystal workpiece surfaces machined by ultra-precision fly cutting. Materials.

[7] An C., Feng K., Wang W., Xu Q., Lei X., Zhang J., Yao X., Li H. (2021). Interaction mechanism of thermal and mechanical field in KDP fly-cutting process. Micromachines.

[8] Pang Q., Shu Z., Kuang L., Xu Y. (2021). Effect of actual frequency features generated in machining process on the temperature and thermal stress of potassium dihydrogen phosphate crystal. Mater. Today Commun..

[9] Liu Q., Liao Z., Cheng J., Xu D., Chen M. (2021). Mechanism of chip formation and surface-defects in orthogonal cutting of soft-brittle potassium dihydrogen phosphate crystals. Mater. Des..

[10] Zhang Y., Hou N., Zhang L. (2019). Understanding the formation mechanism of subsurface damage in potassium dihydrogen phosphate crystals during ultra-precision fly cutting. Adv. Manuf..

[11] Yang Y., Ji Y. (2020). Experimental study on grinding damage control of optical materials. Diam. Abras. Eng..

[12] Chen H., Xu Q., Wang J., Li P., Yuan J., Lyu B., Wang J., Tokunaga K., Yao G., Luo L. (2022). Effect of surface quality on hydrogen/helium irradiation behavior in tungsten. Nucl. Eng. Technol..

[13] Qu M., Jin T., Xie G., Cai R. (2020). Developing a novel binderless diamond grinding wheel with femtosecond laser ablation and evaluating its performance in grinding soft and brittle materials. J. Mater. Process. Technol..

[14] Qu M., Xie G., Jin T., Cai R., Lu A. (2019). Realization of high efficiency and low damage machining of anisotropic KDP crystal by grinding. Precis. Eng..

[15] Yin Y., Zhang Y., Dai Y., Xiao Q., Tie G. (2018). Novel magneto-rheological finishing process of KDP crystal by controlling fluid-crystal temperature difference to restrain deliquescence. CIRP Ann. Manuf. Technol..

[16] Shi F., Qi X., Dong W., Zhu Z. (2019). Improvement of surface laser damage resistance of KDP crystal under combined machining process. Opt. Eng..

[17] Gao W., Wei Q., Ji J., Sun P., Ji F., Wang C., Xu M. (2019). Theoretical modeling and analysis of material removal characteristics for KDP crystal in abrasive-free jet processing. Opt. Express.

[18] Gao H., Wang B., Guo D., Li Y. (2010). Experimental study on abrasive-free polishing for KDP crystal. J. Electrochem. Soc..

[19] Cheng Z., Gao H., Liu Z., Guo D. (2020). Investigation of the trajectory uniformity in water dissolution ultraprecision continuous polishing of large-sized KDP crystal. Int. J. Extrem. Manuf..

[20] Wang X., Gao H., Yuan J. (2020). Experimental investigation and analytical modelling of the tool influence function of the ultra-precision numerical control polishing method based on the water dissolution principle for KDP crystals. Precis. Eng..

[21] Wang X., Gao H., Chen Y., Guo D. (2016). A water dissolution method for removing micro-waviness caused by SPDT process on KDP crystals. Int. J. Adv. Manuf. Technol..

[22] Chen Y., Gao H., Wang X., Guo D., Liu Z. (2018). Laser induced damage of potassium dihydrogen phosphate (KDP) optical crystal machined by water dissolution ultra-precision polishing method. Materials.

[23] Gutmann R., Price D., Neyrick J., Saino C., Permana D., Muraka S. (1998). CMP of copper-polymer interconnect structures. CMP-MIC Conf..

[24] Mullany B., Byrne G. (2003). The effect of slurry viscosity on chemical-mechanical polishing of silicon wafers. J. Mater. Process. Technol..

